# Sulphated glycosaminoglycans support an assortment of planarian rhabdite structures

**DOI:** 10.1242/bio.024554

**Published:** 2017-03-16

**Authors:** Matthew J. Hayes

**Affiliations:** EM Unit, UCL Institute of Ophthalmology, 11-43 Bath Street, London EC1V 9EL, UK

**Keywords:** Planarian, Turbellarian, Rhabdite, Glycosaminoglycan, Tear fluid, Mucus

## Abstract

Planaria are soft-bodied, bilateral flatworms of the phylum Platyhelminthes. They are covered in cilia and use ciliary-gliding to traverse the substratum while hunting. Their body surface is covered in a layer of viscous slime primarily derived from specialised secretory granules known as rhabdites. The slime must somehow stay associated with the surface of the animal in aqueous environments whilst also lubricating the interface of the animal and the surfaces over which the animal moves. The slime prevents damage to the animal's soft body and also contributes to adhesion to the substratum. In order to gain insight into how it might achieve these diverse functions, we performed electron microscopic examination of the slime's structure. Analysis of two freshwater flatworms from the UK *Schmidtea polychroa* (Schmidt, 1861) and *Polycelis tenuis* (Ijima, 1884) revealed a high level of organisation of the slime layer and a variety of ejected slime structures. We show that these structures are rich in sulphated glycosaminoglycans (sGAGs). Most of these (269 of 285 examined) appear to be topologically closed spheroids that we name ball-GAGs. Another class appears to burst to release flower- and star-like clusters which adhere to motile cilia. We also observe fibrous nets that are associated with entrapped bacteria. Examination of the structure of rhabdites ejected onto a porous surface suggests a mechanism by which their structure allows them to both bind to the porous surface and provide a smooth layer over which the animal could glide. Such sGAG-based structures might provide models for the design of artificial biomimetic replacements for tears, saliva, bio-compatible lubricants or drug-delivery vehicles.

## Abstract (precis)

**Summary:** We show that the slime secreted by planarian flatworms is supported by a complex meshwork of sulphated polysaccharides. These form an assortment of geodesic structures. Similar structures could be used to provide novel biomimetic drug delivery vehicles.

## INTRODUCTION

Planarians are bilaterally symmetric acoelomate, triploblastic metazoans of the phylum Platyhelminthes (flatworms). They have no respiratory or circulatory systems and no anus, sucking up prey through a mouth situated at the end of a trunk-like everted pharynx or ‘proboscis’ that usually emanates from the mid-ventral part of the body. They are commonly found in freshwater streams and ponds where they prey upon smaller invertebrates. Planarians are covered in ciliated epithelium and by a secretion of mucus-like slime that has been implicated in innate immunity, ciliary gliding, substrate-adhesion, predator avoidance and prey capture (Pedersen, 1953, 1963; Martin, 1978). Given the physiological similarities between the planarian epithelium and ciliated epithelia of mammals, they have been considered a useful model organism for pathologies involving inefficient mucus transit such as asthma, chronic obstructive pulmonary disease (COPD), emphysema, bronchitis and cystic fibrosis (Bocchinfuso et al., 2012).

**Fig. 1.**
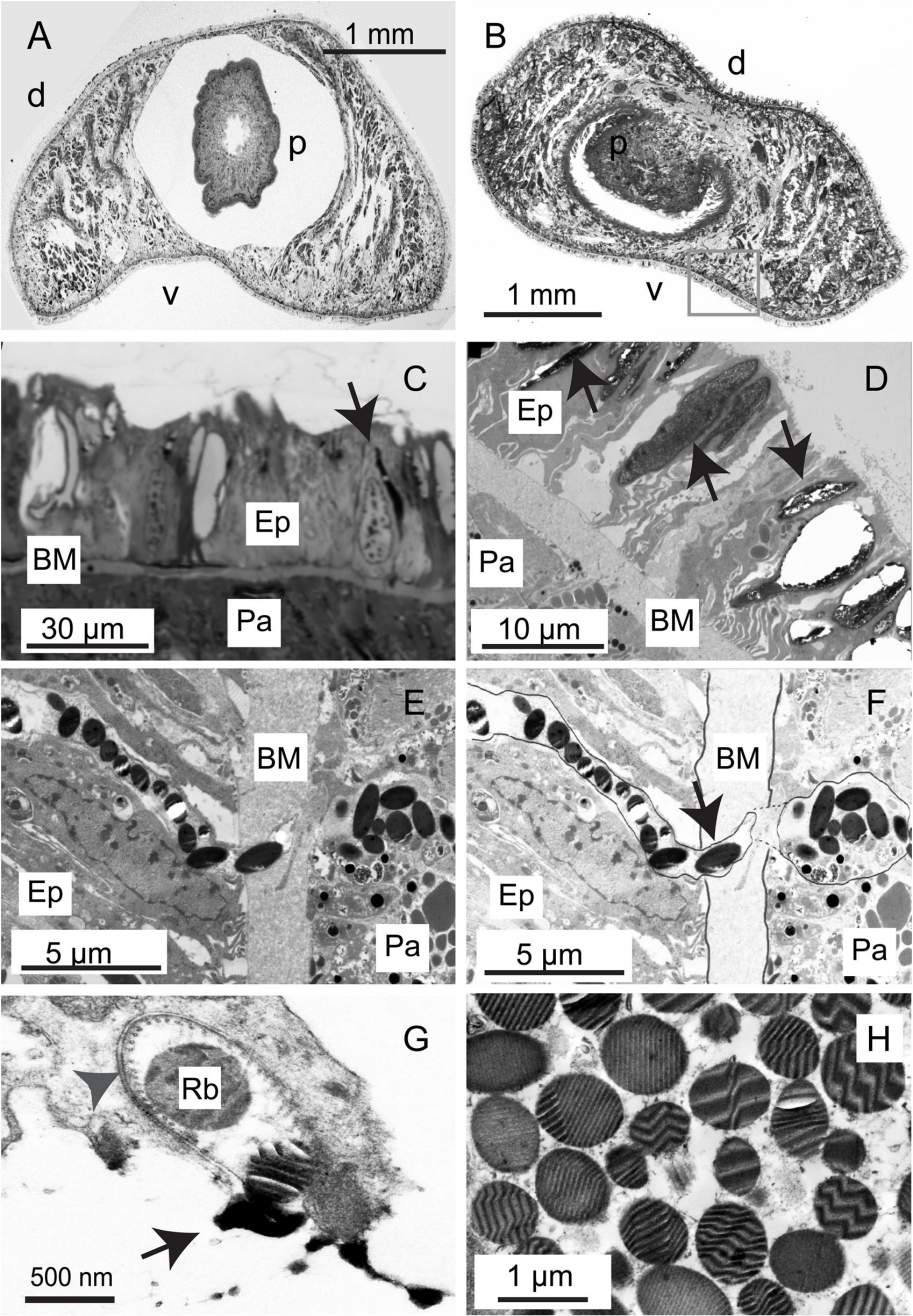
**Distribution of epithelial and adenal rhabdites in *P. tenuis* and *S. polychroa.*** (A) A low-power, bright field image of a Toluidine Blue-stained semi-thin section through the middle of the body of *P. tenuis*: dorsal (d) and ventral (v) surfaces are indicated as well as the proboscis (p). (B) A low-power image of *S. polychroa*: dorsal (d), ventral (v) surfaces are marked as well as the proboscis (p)*.* The epidermis and underlying parenchyma are demarcated by the grey box. (C) A close up of the epidermal epithelium (Ep) and parenchyma (Pa) separated by a prominent basement membrane (BM). It is possible to see un-ejected epithelial rhabdites (black arrow). (D) Structure of the epithelium and underlying parenchyma revealed by TEM. The epithelial rhabdites are visible (black arrows). (E) ‘Adenal rhabdites’ derived from parenchymal cells. (F) The same image as in E annotated for clarity. The tubule down which the adenal rhabdites migrate passes through the basement membrane (black arrow). (G) An adenal rhabdite (Rb) (black arrow) in the process of being ejected through the epidermis from a microtubule-lined tubule (grey arrowhead). (H) Striated adenal rhabdites in a parenchymal cell.

Planarians are members of the class Rhabditophora, characterised by the possession of laminar granules or rhabdites. Planarian slime is derived from these rhabdites, and historically, histological examination of these has been used to establish taxonomy amongst groups (Smith III et al., 1982). Rhabdites fall into two broad categories. One group are present in the outermost epidermal layer. These are sometimes termed ‘triclad-type’, ‘mucoid bodies’, or epithelial rhabdoids (Smith III et al., 1982). They are large (>5 µm before secretion and >15 µm afterwards) and are the subject of this paper (Fig. 1A-D). A second group are generated by cells in the inner, parenchymal cell layers and are released at the surface through tubular, microtubule-lined necks. These are smaller in size (0.4-0.7 µm), are often highly striated, and are sometimes referred to as ‘adenal rhabdites’ (Fig. 1E-H). A further category comprises those rhabdites generated by the duo-gland adhesive and release system (Tyler, 1976, 1988; Whittington and Cribb, 2001). This fascinating organ is formed of a number of specialised cell types and forms a gland which facilitates transient adhesion by the secretion of adhesive glue (small 0.4-0.7 µm long rhabdites) and an agent that counteracts this adhesion. This type of gland can be identified by the presence of modified epithelial cells (anchor cells) and a crown of surrounding cilia.

Recently, a proteomic analysis of planarian slime demonstrated that it shared significant similarity to nasal mucus, olfactory mucus, cervical mucus, and tear fluid (Bocchinfuso et al., 2012). Histological studies revealed that the rhabdites and related organelles contain little lipid but some are cyanophilic, staining with Alcian Blue, and are thought to contain neutral and slightly acidic glycosaminoglycans (Pedersen, 1953, 1963; Bowen et al., 1975).

There has been little study of rhabdite structure once extruded, though it is in the extruded state that they are most likely perform their many roles. Rhabdites may remain closely associated with the animal's body or they may be left as a slime ‘trail’. They allow adhesion to surfaces, prey or mates without causing the animal to become ‘stuck’ or interfering with ciliary function, suggesting a degree of control of its stickiness. They must be lubricating and compressible and cover a large surface area. This led us to investigate their shape following extrusion to see if some of this versatility could be explained by their structure.

In this study we used TEM to examine extruded rhabdites from *Schmidtea polychroa* (Schmidt, 1861) and *Polycelis tenuis* (Ijima, 1884). Epithelial rhabdites, adenal rhabdites and duo-gland rhabdites are all present. We identify the epithelial rhabdites by virtue of their greater size.

We also used Cupromeronic Blue to stain sulphated glycosaminoglycans (sGAGs) in the slime layer. We examined the fine structure of these ejected rhabdites both on the surface of the animal and when they are extruded onto a porous surface. We identify an unexpected complexity in the structure of the slime layer and in the arrangement of these sGAGs. Such ‘architectural’ complexity has not been previously identified in other slimes, suggesting it has unique features. These observations may provide useful models for the design of artificial bio-lubricants/wetting agents (such as artificial tears) or for the design of potential drug delivery vehicles.

## RESULTS

### Rhabdites on the surface of planaria

**Fig. 2.**
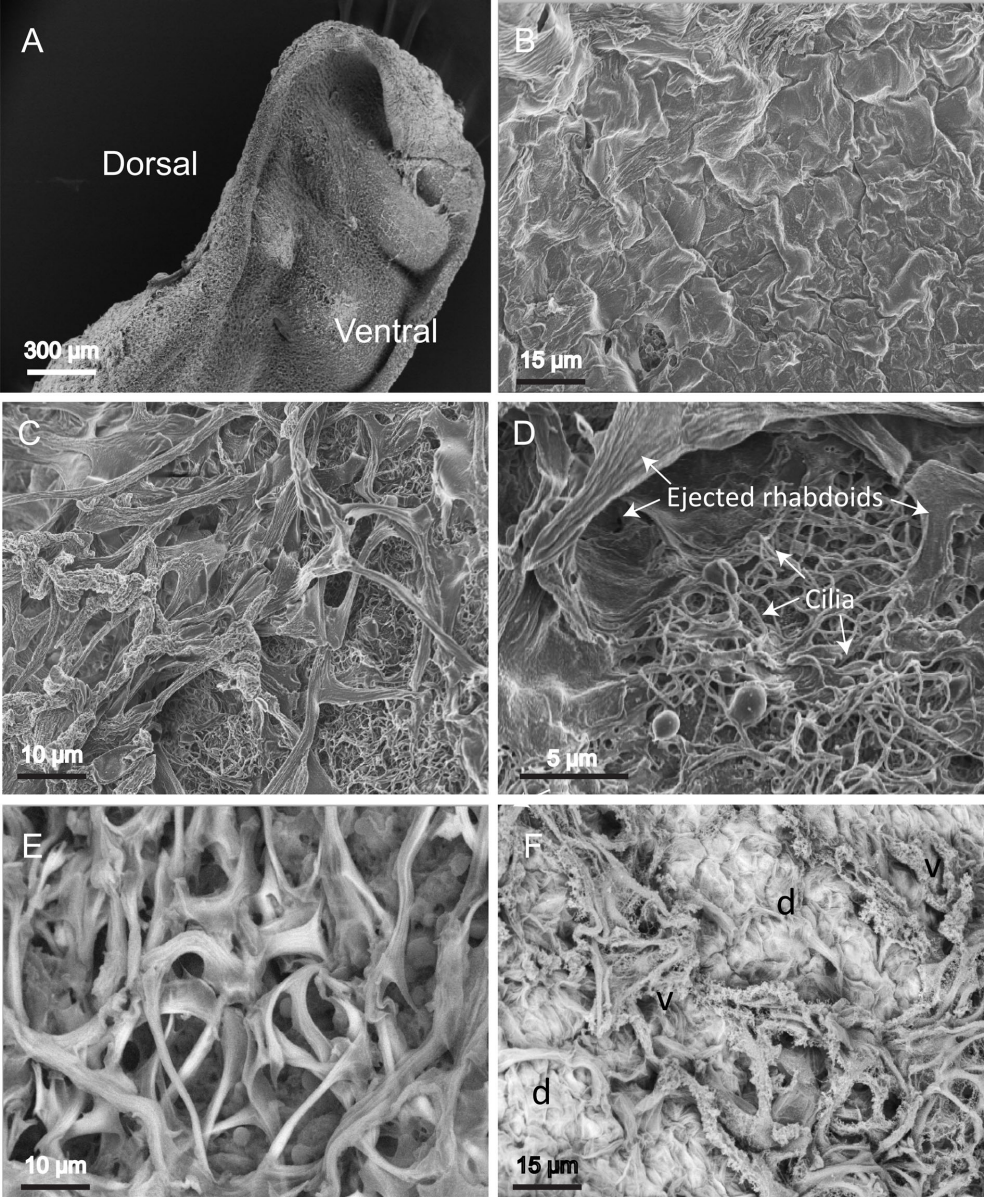
**Rhabdite structure in *P. tenuis* as revealed by SEM.** (A) Low-power image of *P. tenuis* showing the dorsal and ventral surfaces. (B) Close-up of ventral surface showing dense arrays of cilia. (C) Image of dorsal side of *P. tenuis* showing the close-packed, extruded rhabdites piled on top of one another. (D) Close-up, angular, partially folded rhabdites on the ventral surface. The cilia can be seen supporting them. (E) The same surface as in D viewed with the variable-pressure back-scatter detector. (F) A region near the flank of the animal showing both types of rhabdite. Many of the angular rhabdites have burst to reveal their granular internal structure (variable-pressure back-scatter detector). d, dorsal; v, ventral. Samples shown are representative of five specimens examined.

The dorsal and ventral surfaces of the planarian are covered in different types of ejected rhabdite. The dorsal surface (Fig. 2A,B) is mostly covered with a layer of rhabdites that present a smooth, uninterrupted surface. Observed by scanning electron microscopy, they look somewhat like piles of bundles of clothes, one rolling against and on top of the next. The overlapping nature of the objects made it impossible to calculate their dimensions with any degree of accuracy using SEM, but they appear to be larger than 15 µm long and 15 µm wide. The ventral surface (Fig. 2C-E) is more sparsely covered in rhabdites and the supporting cilia can clearly be seen beneath them (Fig. 2D). In *P. tenuis* the ventral rhabdites appear long and thin (32.6±8.6 µm long *n*=100), angular and petal-like by back-scatter SEM (Fig. 2E). In the lateral zone both dorsal type (d) and ventral type (v) of rhabdite can be seen (Fig. 2F).

### Rhabdites unfold into topologically closed spheroids

**Fig. 3.**
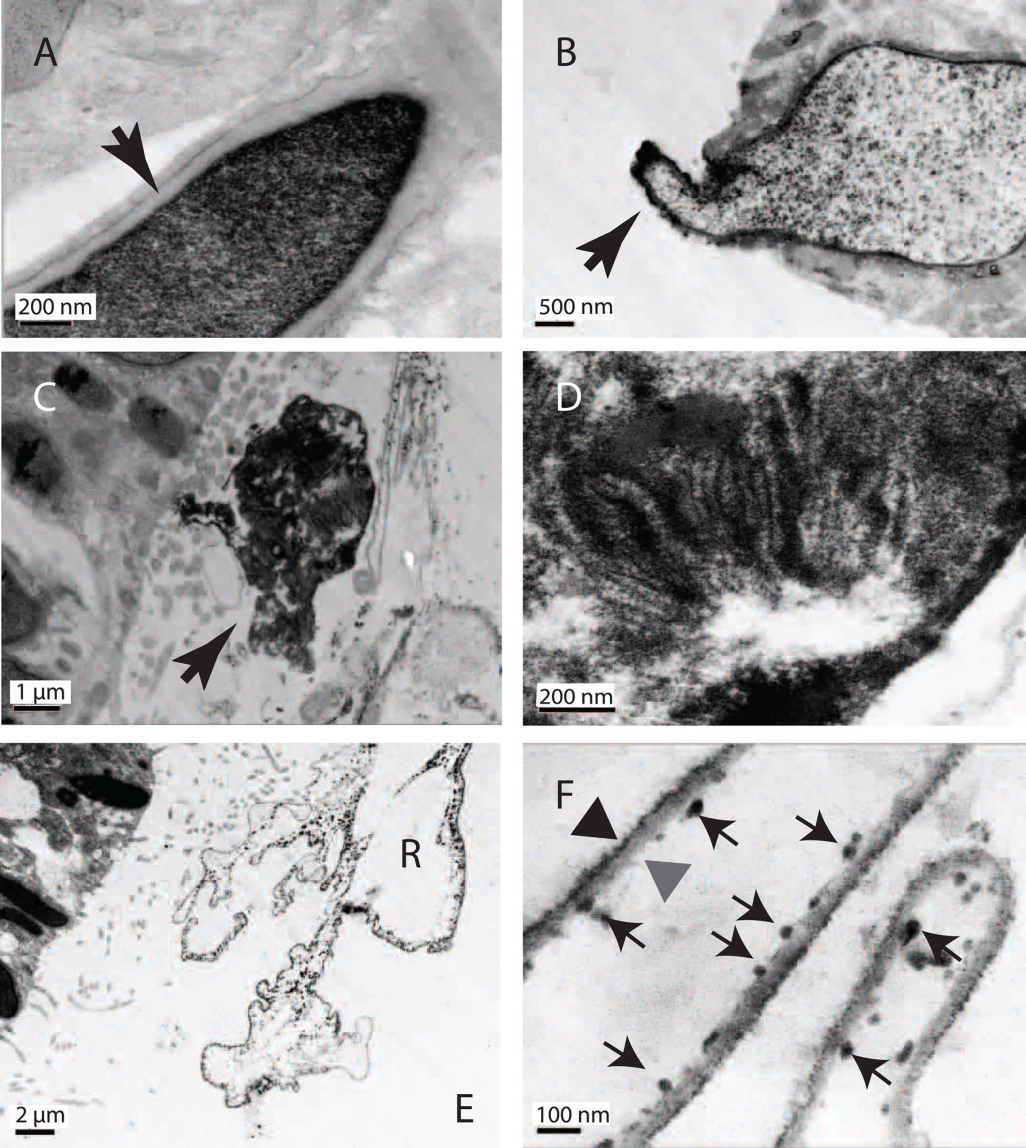
**Epithelial rhabdite structure in *P. tenuis* as revealed by conventional TEM.** (A) Rhabdites appear as dense granular, pointed ellipsoids surrounded by a less darkly staining density at the ‘outer membrane’ (black arrow). (B) Rhabdite undergoing expulsion from the surface. The granule is swollen and the contents appear as punctate densities. The ‘outer membrane’ is intact as it passes out of the cell (black arrow). (C) A dense extruded rhabdite (black arrow). (D) Close-up revealing folded structure in the arrangement of internal components. (E) A large rhabdite held away from the surface epithelium of the planarian by cilia. R, rhabdite. (F) Close up of an expanded rhabdite showing two layers (black and grey arrowheads) decorated by internal dense granules (small arrows). Samples shown are representative of five specimens examined.

Conventional TEM reveals *P. tenuis* rhabdites in the outer ventral epidermal layer to be pointed, elongated granules (8.5±4.5 µm long, *n*=100). They have a cortical limiting domain which has been described as a ‘membrane’ (Smith III et al., 1982), and contain what appear to be uniformly granular contents (Fig. 3A). Swelling may occur inside or outside the epidermis. In some cases rhabdites appear to be in the process of swelling (Fig. 3B) (the granular staining is more diffuse) and the tip of the rhabdite can be seen protruding from the cell. In the case of fully ejected rhabdites the previously homogeneous material is revealed to be folded into a complex pattern (Fig. 3C,D). Fully ejected rhabdites remain associated with cilia (Fig. 3E), but are held clear of the surface of the animal. At this low magnification it is possible to identify different types of ‘outer membrane’ (OM) showing a range of electron densities and associations with particulate material. A close up of one region reveals a multi-laminate structure of larger, electron-dense outer filaments surrounding a thicker, less dense layer (Fig. 3F, black and grey arrowheads). The ‘inside’ of the expanded rhabdite is decorated with larger electron-dense aggregates of material (small black arrows) (averaging 32±6 nm in this example). It is notable that these are always on one side of the OM and in certain parts of the expanded rhabdite the different fibrous layers have separated; suggesting they are not tightly associated. Such structures were present in all the planaria we examined. Serial sectioning of six expanded rhabdites was carried out to establish if they were topologically closed. We rarely saw breaks in the membrane and all aggregates were on the inside. These observations lead us to conclude that many rhabdites are closed spheroids (or at least are initially topologically closed when ejected) (Fig. S1).

### The rhabdite structure is maintained by sGAGs

**Fig. 4.**
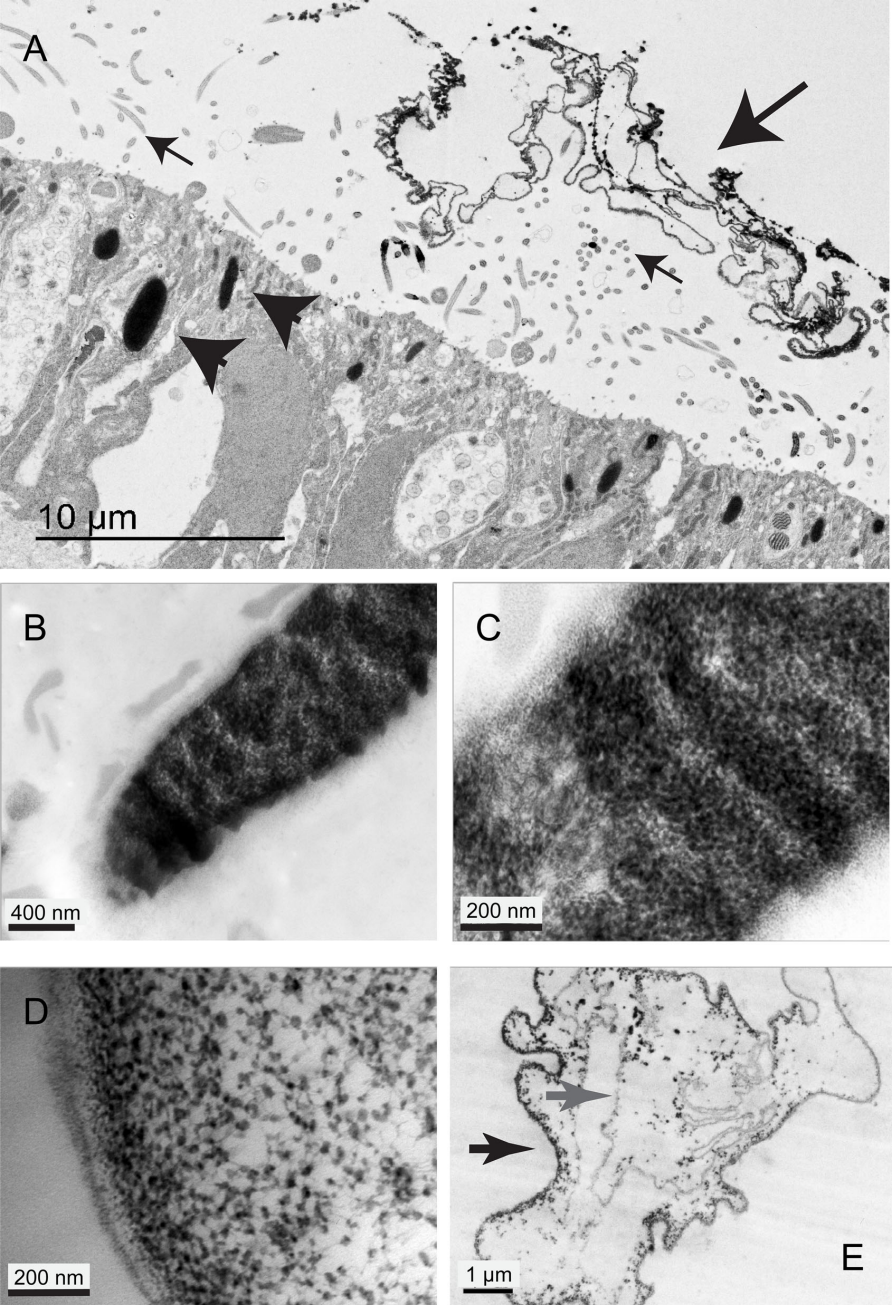
**Rhabdite glycosaminoglycans can be stained by Cupromeronic Blue. By TEM they show a complex variety of ultrastructures.** (A) Rhabdites on the dorsal surface. Ejected rhabdites (large black arrow) stain darkly with Cupromeronic Blue, indicating they contain sulphated GAGs. They are held away from the surface of the animal by cilia (small arrows). Un-ejected rhabdites are clearly seen in the epithelium (black arrowheads). (B) Close-up of rhabdite stained with Cupromeronic Blue. The sulphated glycosaminoglycan (sGAG) material is arranged in patches and layers. (C) A close-up of B showing how the patchy material is composed of many punctae connected by filaments. (D) Close-up of a region where the rhabdite has started to expand. (E) This ejected rhabdite is a complex, multi-layered structure with thicker, more darkly-staining elements (black arrow) and thinner layers (grey arrow). The darker, punctate material appears to have differing affinities for the different layers. Samples shown are representative of ten specimens examined.

Rhabdites from *P. tenuis* and *S. polychroa* were stained with the cationic phthalocyanin dye, Cupromeronic Blue (Scott, 1980). This stain can be used in electron microscopy to label highly charged sulphated glycosaminoglycans. In this protocol lipids and proteins appear very pale as only tungsten salts are used as a counter-stain. Ejected rhabdites stain intensely, suggesting they contain sGAGs. To confirm the specificity of the staining protocol samples were treated identically but without the addition of the Cupromeronic Blue stain. In this case the rhabdites, both ejected and internal, appeared far more electron lucent. Filamentous structures were barely visible even following counter-staining with Reynold's lead citrate (Fig. S2). Rhabdites which have not been ejected exhibit a convoluted pattern of staining (Fig. 4A). This reveals a folded matrix, composed of individual dense aggregations connected by filamentous material (Fig. 4B-D). The ejected and expanded rhabdites maintain their intense staining and both aggregates and ‘membranous’ structures stain strongly, indicating that both contain significant amounts of sGAGs (Fig. 4E). The OM sometimes separates into strongly positive-staining layers (black arrow) and less positive layers (grey arrow). This may reflect different degrees of sulphation of the GAGs in the more electron lucent layers or indicate there are fewer GAGs (and perhaps a higher proportion of protein) in this domain.

### Some swelling rhabdites disintegrate to release sticky contents

**Fig. 5.**
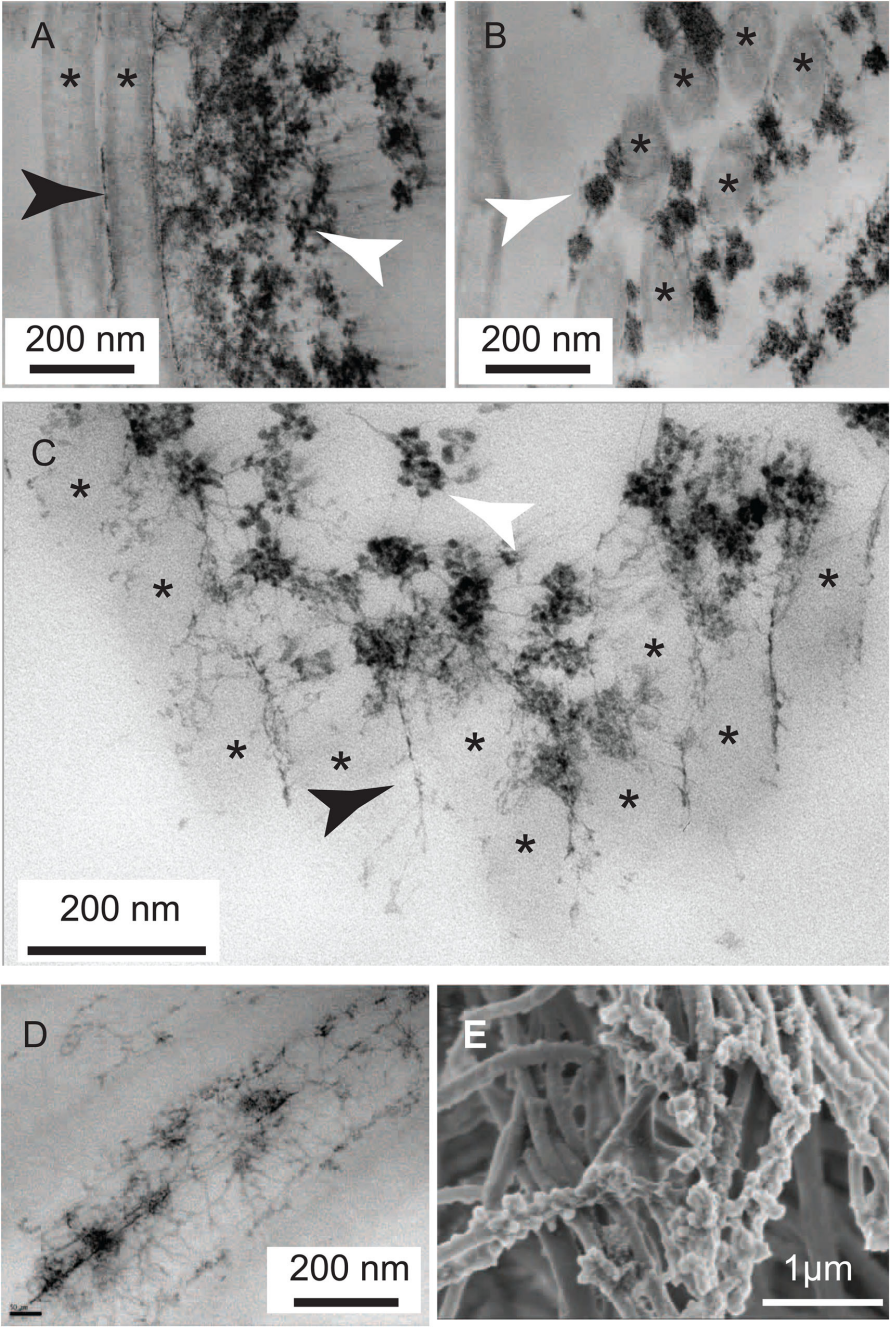
**Upon rupture some rhabdites retain structural elements which allow them to attach to cilia.** (A) Sample stained with Cupromeronic Blue (but only counter-stained with tungstate) reveals flower-like clusters of punctate structures (white arrow) held together by fine filaments which are themselves tethered to the cilia (*) by means of adherent sGAG filaments (black arrow). (B) A similar cluster as in A, viewed *en face*. (C) A close up of sGAGs on cilia (*) showing aggregates (white arrowhead) and interconnecting filaments (black arrowhead). (D) A close up of sGAG filaments stuck to the surface of a cilium. (E) These structures are also clearly revealed on the surface of cilia by scanning electron microscopy. Samples shown are representative of ten specimens examined.

Although most rhabdites on the surface of the animal (93% *n*>200) appear as topologically closed surfaces (often appearing stacked one upon the other), a subset of ventral-surface rhabdites rupture and release their granular contents. Upon staining with Cupromeronic Blue it was apparent that the granules are themselves flower-like aggregates of small (11±3 nm) particles held together by filamentous sGAG threads. Upon release, these particles decorate the cilia, binding to them by means of long, very thin, web-like sGAG threads. Those from *S. polychroa* were small aggregations that ultimately appeared to disintegrate into tangles of sGAG (Fig. 5A-E).

### A variety of rhabdite structures

**Fig. 6.**
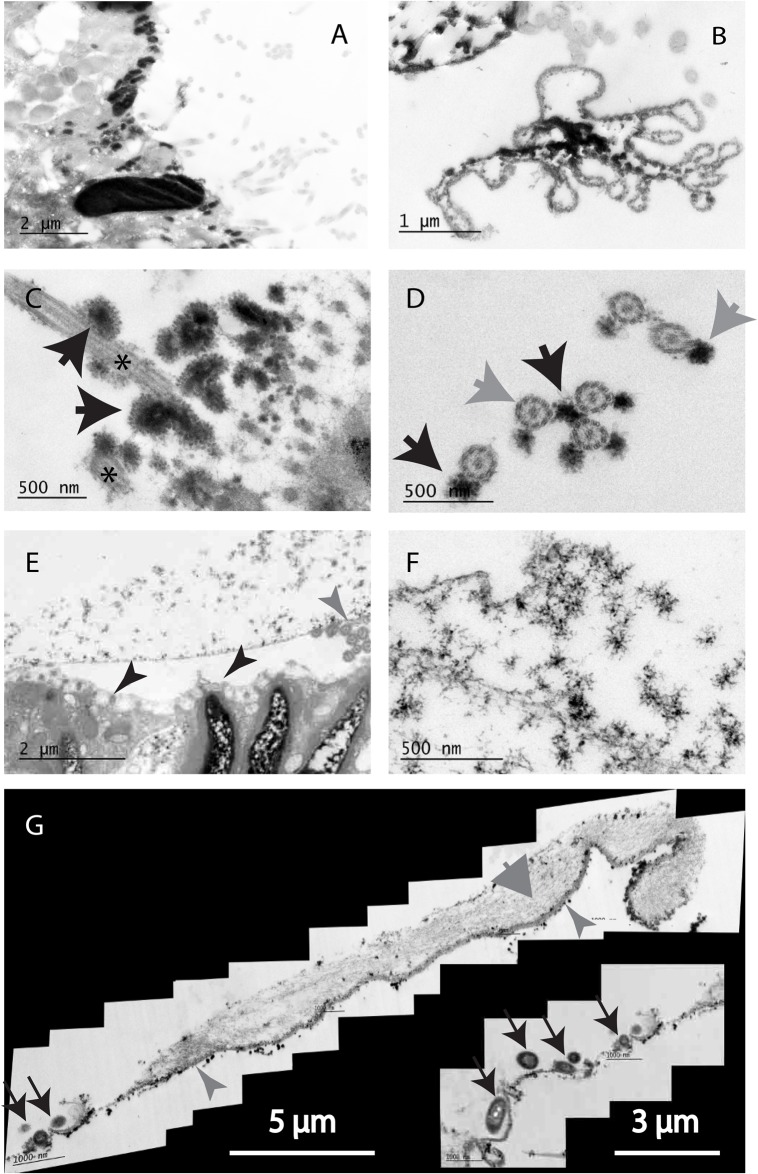
**Different species demonstrate a range of different sGAG-containing structures.** (A) Epithelium of *S. polychroa* showing both large and small strongly-staining rhabdites: nb. the spiral structure of the large rhabdite. (B) An ejected *S. polychroa* rhabdite demonstrating a thick, fibrous ‘outer membrane’ and large internal fibrous granules. (C) Large granules associated with cilia (*). They are composed of a dense core surrounded by many smaller particles attached to the core by thin filaments (black arrowhead). (D) A similar region as in C seen *en face* showing granules (black arrow) cilia (grey arrow). The ‘9+2’ structure of the microtubules in the cilia is clearly visible. (E) A large swollen rhabdite from a small unidentified planarian held clear of the epithelial surface by cilia (grey arrow) and microvilli (black arrows). (F) The granular contents of this rhabdite are a loosely associated star-shaped aggregate. (G) A large, ruptured rhabdite with a thick ‘outer membrane’ (small grey arrowheads) associated with a complex fibrous sGAG-containing ‘net’ (large grey arrowhead). Such ‘nets’ were sometimes associated with adherent bacteria (black arrows) (G and insert). Samples shown are representative of 15 specimens of *S. polychroa* examined.

Rhabdites from *P. tenuis* had relatively thick ‘outer membranes’ and contain large ‘fluffy’ aggregates with a central dense core of small particles surrounded by smaller satellite particles connected by thin filaments (Fig. 6A-D). In another unidentified small planarian (possibly also *P. tenuis*) we identified large expanded rhabdites which contained clusters of open, star-shaped filaments (Fig. 6E,F). We also observed large, net-like expanded rhabdites which were sometimes associated with bacteria (Fig. 6G).

### Structure of rhabdites secreted onto a porous surface

**Fig. 7.**
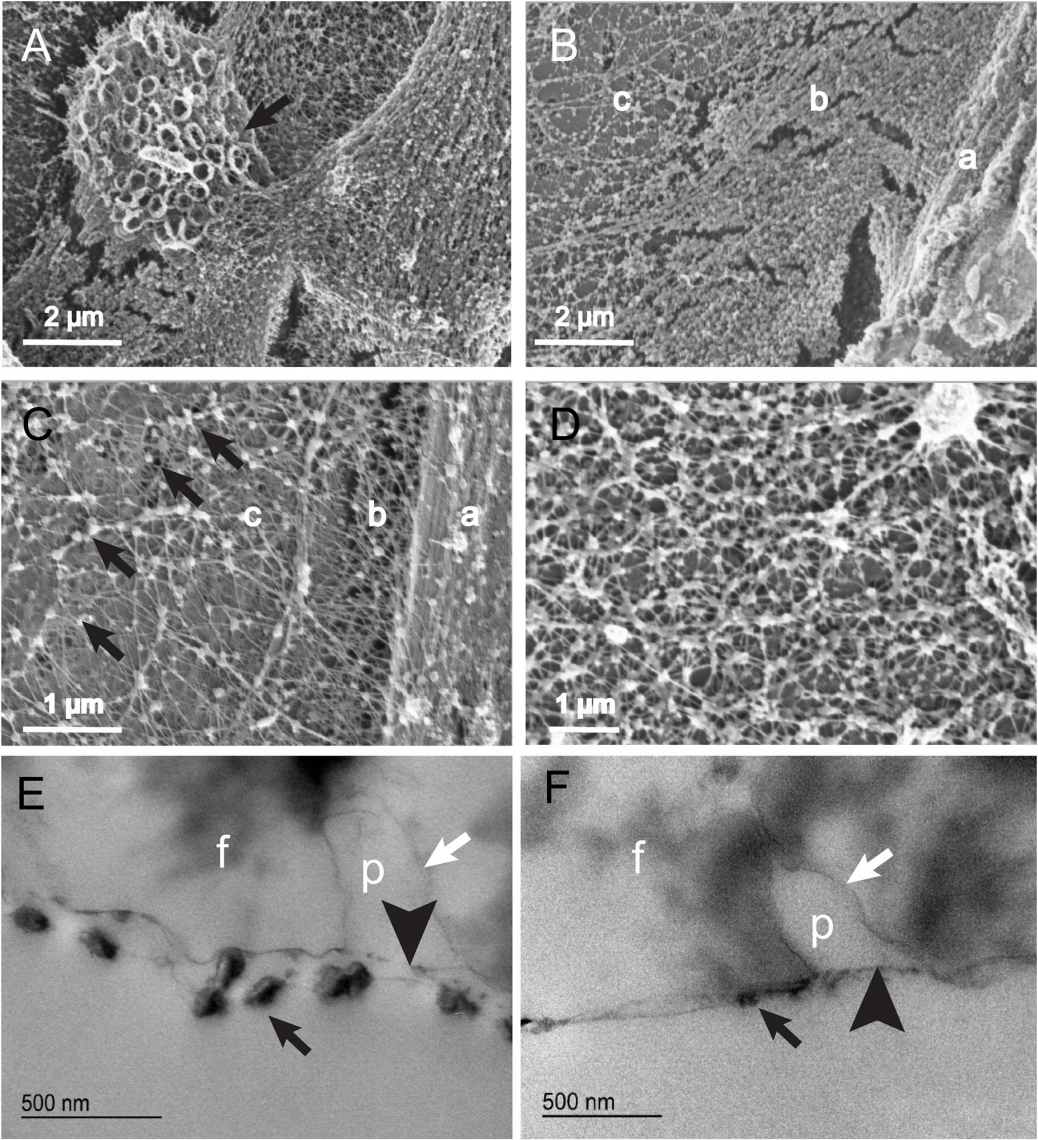
**Structure of rhabdites secreted onto support surfaces.** (A) By scanning electron microscopy the ejected rhabdites that have attached to a porous surface can been seen. Newly ejected rhabdites (black arrow) appear dense, but occasionally exhibit complicated folds (such as the rings observed in this example). (B,C) The rhabdite unfolds and opens out onto the surface, becoming progressively less dense (a to c). In C the pores of the filter can just be seen underneath the net of the rhabdite (black arrows). (D) A close up of a flat part of the filter covered in a network of rhabdite. Spheroidal aggregates are separated by radiating filaments. (E,F) Cupromeronic Blue staining of TEM sections through these filters reveal the aggregates and filaments contain sGAGs. The outer layer of the deposited ball-GAG expands into the underlying stratum [in this case the pores of the milipore filter (white arrows)]. The inner later, carrying the dense GAG aggregates forms a flat layer that does not penetrate into the pores of the filter (black arrowhead). Black arrows indicate sGAG aggregates; f, filter; p, pore. Samples shown are representative of 6 specimens.

Animals were allowed to crawl for 12 h over a porous filter. Any slime ‘trail’ they left behind after this time was fixed and stained, and examined by both scanning and transmission electron microscopy. SEM revealed some ejected rhabdites consist of dense balls of material, sometimes folded into complex patterns (Fig. 7A). Most appeared as flattened sheets of varying density, sometimes overlapping, but mostly abutting one another on the surface of the filter. The rhabdite most intimately associated with the filter was usually that with the most ‘open’ structure. Progressively more densely packed structures were more loosely attached to the surface, or lay on top of the more open ones, suggesting that the rhabdites expanded or spread as they adsorbed to the porous surface. Close examination of these revealed that they represented punctate aggregates of material connected by multiple very thin filamentous connections (Fig. 7A-D).

TEM revealed the mesh to be at least partly composed of sGAGs, Cupromeronic Blue staining dense aggregates being separated by fine filaments. In some places the net was maintained as a double-thickness layer (presumably representing a laterally flattened yet topologically closed rhabdite), in others they appear to have ruptured, only one side of the rhabdite being present. In all cases, it was apparent that the outer layer of the rhabdite had migrated into the microporous spaces of the membrane, forming an intimate association with it, whilst the inner later remained flat (Fig. 7E,F).

## DISCUSSION

Biology provides many fascinating examples of mucus-like slimes. These have multiple functions: lubricating, protecting, preventing desiccation, trapping food, hindering infection and forming bio-adhesives to name a few (Denny, 1989; Smith, 2002; Fudge et al., 2015). Each application has particular requirements in terms of its viscoelastic, adhesive, or rheological properties. A number of recurrent biochemical themes have been identified that provide some of this diversity. Biological slimes are hydrogels containing a high proportion of water (often more than 90%). There are often both protein and polysaccharide components and these may be supplemented by inorganic components (Werneke et al., 2007). Slimes such as vertebrate mucus are formed of massive, elongated glycoproteins and form non-crosslinked gels by means of the entangling of their oligosaccharide side chains (Silberberg and Meyer, 1982; Bansil and Turner, 2006). In other cases the slime may predominantly comprise megadalton-sized cross-linked mucopolysaccharides. Echinoderm podia and tentacles adhere to surfaces using such GAG secretions (Flammang, 1996). The dominant protein species may also be relatively short, highly cross-linked proteins (Denny, 1989; Smith 2002). Some organisms are able to produce a range of slimes with different properties. Variation in the biochemical content of mucus secretions has been noted in gastropods, for example (Smith et al., 1999; Smith and Morin, 2002), and is thought to alter its adhesive properties and physiological function. The majority of studies have looked at biophysical or biochemical properties of slimes and have proposed models of function based upon these data.

Planarian slime is derived from specialised secretory granules called rhabdites. Variation in rhabdite size and biochemical content (using histological dyes) between species and in different parts of the animal has been noted (Bowen et al., 1975; Smith et al., 1982) but there have been no in-depth studies of the slime's structure.

sGAGs have been putatively identified in Platyhelminthes in whole-organism preparations (Yamada et al., 2011). They include heparan sulphate (a polymer of repeated disaccharide units of glycuronic acid or iduronic acid and 2-deoxy-2-sulphamido-α-D-glucopyranosyl-6-O-sulfate) and chondroitin sulphate (a polymer of repeated disaccharide units of glucuronic acid or iduronic acid and N-acetyl-D-galactosamine). This, and histological studies, have suggested that large mucopolysaccharides such as GAGs could contribute to rhabdite-derived slime.

In this study we have confirmed the presence of sGAGs in rhabdites using the sGAG stain Cupromeronic Blue, and show that they are not homogeneously distributed in the slime.

It is difficult to compare our observations of slime structure in planarians with those from other organisms because we have only employed imaging-based methods to identify ‘gross’ structure and this has not been widely applied to other organisms. However, our data indicate that planarian slime is rather different from that of other organisms. It appears that nets and ‘membranous’ complexes of sGAGs impose order on the slime secretions and these can persist, potentially for many hours.

The rhabdites unfold to form hollow, spheroid meshes (ball-GAGs) and star- and flower-like aggregates, sometimes suspended within the ball-GAG, and sometimes released to bind freely with the cilia-covered surface of the animal. Some ball-GAGs show evidence of being multi-layered: different densities we observed in Cupromeronic Blue-stained ball-GAGs perhaps indicating highly sulphated and less-sulphated domains or sGAG-rich and more protein-rich ones. This may be functionally analogous to the double-network gels generated by certain terrestrial slugs (Wilks et al., 2015); a duel-component meshwork which has emergent properties greater than the sum of its parts.

The form of the epithelial rhabdoid when secreted onto a rough surface also highlights the potential of spatial separation of components. The dense granules appear to open out and spread over the surface, providing a ‘carpet’ for the animal to crawl over. The ‘single fibrillary layer’ or ‘membrane’ described by other authors is rather a loosely-bound multi-lamellar structure. The presented surface is a dense array of criss-crossed sGAG-containing filaments connected or decorated by aggregates of sGAG-containing material. One sGAG layer, by adapting its shape to the surface, maximizes the substrate-sGAG interface and thus may optimise binding to it. The other sGAG layer provides a flat, smooth surface over which the animal could move; but one that is still attached to the former, adaptive sGAG and is perhaps ‘lubricated’ against it by the sGAG aggregates that constitute the hydrogel that sometimes lines the thin cavity between them.

Our observations are preliminary and speculative and need to be followed up by rigorous biochemical/biophysical experimentation; but they appear to represent an example from nature of a ‘configured hydrogel’ where spatially separated biochemical and biophysical content and architectural form all contribute to function.

The sGAG's contribution to the structure of the ejected rhabdite may provide a scaffold for their proteinaceous content and determine how and when this is deployed. Even if some of the protein constituents are common to both planaria and vertebrates, the structured sGAGs we observe might be expected to confer very different properties. In some cases, sGAGs appear as intricately folded OMs or ‘envelopes’ which contain clustered cargo, which itself appears to be tethered together by other sGAGs. One might speculate upon a situation where one protein was isolated from others by association with different elements of the three dimensional architecture of the unfolded rhabdite. One protein might be associated with the centre of a ‘flower-like’ aggregate, whilst others might only be found at the tips of the radiating projections (such as those seen in Fig. 7C or F). In another case, one protein might be associated with freely diffusing aggregates, whilst others might be linked to the net-like OMs. In this way a protein that contributed to the adhesive properties of the slime could be separated from another that was capable of degrading it, thus reducing adhesion.

The fibrous outer layer of the rhabdite has been described as a ‘membrane’ but this is an inappropriate histological description of something that at the electron microscope level appears as a tightly-folded mesh-like bag of sGAGs. For obvious reasons have called this structure a ball-GAG, though the Russian word ‘avoska’ appeals as it pertains to tightly folded net-bags that have a range of uses. Sometimes we identified examples that had torn and released their contents. The sticky aggregates released then associate with the motile cilia by means of sGAG filaments. This time-dependent, disaggregation of the structure may allow the rhabdite to exhibit different properties at different times.

Sulphated glycosaminoglycans have a range of potential applications in medicine (Köwitsch et al., 2017). Hydrogels, such as those containing the non-sulphated GAG hyaluronic acid (a naturally occurring biomaterial) have been shown to promote drug dwell-time on the cornea, for example. It is thought that this occurs by maximising so-called ‘mucoadhesion’, the association of the supported drug with the mucus layer adherent to the corneal epithelium (Greaves and Wilson, 1993; Ludwig, 2005).

We propose that sGAG-based, structurally complex synthetic ‘configured hydrogels’ with 3D-order and spatial segregation modelled on those we have identified in the planaria could be used for drug delivery, to the front of the eye for example, or to ciliated epithelia.

## MATERIALS AND METHODS

Animals were collected from the wild in Surrey, UK. Thirty specimens of *Schmidtea polychroa* (Schmidt, 1861) and twenty-five of *Polycelis tenuis* (Ijima, 1884) were examined over a two year period.

### Transmission electron microscopy

Planaria were fixed in cold (4°C) Karnovky's fixative (2% paraformaldehyde and 2.5% glutaraldehyde in 0.08 M cacodylate buffer). They were not washed prior to fixation in order to preserve the slime associated with their bodies in its native state. They were then washed three times in phosphate buffer and osmicated with 1% osmium tetroxide in ddH_2_O for 1 h. Samples were then washed 3×10 min in ddH_2_O and dehydrated with a series of alcohols: 30%, 50%, 70%, 90%, 3×100% and 2× propylene oxide (at least 20 min in each). They were infiltrated with 50% propylene oxide: 50% araldite resin overnight and with several changes of 100% resin the next day. Resin blocks were cured at 60°C overnight. Sectioning was done using a Leica Ultracut UCT microtome. Sections were sometimes counter-stained with Reynold's lead citrate. Sections were viewed on a JEOL 1010 TEM (JEOLUSA, MA, USA).

### Toluidine Blue staining and histology

650 nm ‘semi-thin’ sections were cut from araldite-embedded specimens and dried-down onto glass slides. They were then stained with 0.5% Toluidine Blue in 2% di-sodium tetraborate and imaged using differential interference contrast on a Zeiss 710 confocal microscope.

### Critical-electrolyte-concentration Cupromeronic Blue staining

Planaria were fixed in Karnovsky's fixative as described above for 30 min then washed three times in 25 mM sodium acetate buffer pH 5.7 before being stained in Cupromeronic Blue stain (25 mM sodium acetate pH 5.7, 0.2 M MgCl_2_, 0.05% Cupromeronic Blue) overnight at 4°C. At this electrolyte concentration the station is specific for the highly charged sulphated glycosaminoglycans (Scott, 1980). The sample was then washed 3×10 min in 25 mM sodium acetate, 3×10 min in aqueous 0.5% sodium tungstate and dehydrated in 50% ethanol/50% 0.5% sodium tungstate, 70%, 90%, 3×100% ethanol and 2× propylene oxide and then embedded/sectioned as above. Sections were sometimes counter-stained with 0.5% aqueous uranyl acetate.

### Scanning electron microscopy

Planaria were fixed, osmicated and dehydrated as described above, then further dehydrated by rapid immersion in hexamethyldisilazane (Sigma-Aldrich, USA) for 2 min, then were allowed to dry on a conductive carbon-tab on an aluminium SEM stub. The sample was ‘back-filled’ with silver paint to promote conductivity, allowed to dry for 2 h in a desiccator and platinum-coated (1.5 nm) in a sputter-coater (Cressington 108 auto, Cressington, UK). Samples were examined using a Zeiss Sigma SEM using both in-lens and VP-back scatter detectors (Zeiss, Germany).

### Scanning electron microscopy of extruded rhabdites

Planaria in filter-sterilised pond water were allowed to crawl for 4 h over the surface of polycarbonate membranes in 12 well dishes (both from Costar, NY, USA). The animals were removed and the membranes fixed in Karnovsky's fixative for 4 h. The filters were stained with Cupromeronic Blue as described above to establish if the animals had secreted any ‘trail’. The samples were then dehydrated and either prepared for TEM or SEM.

### Measurements

Dimensions were estimated by measurements recorded from micrographs made using Image J. The figures represent the mean of at least 50 rhabdites±standard deviation. Extruded rhabdites were classified as ‘closed spheroids’ in 2D images if a continuous line could be drawn around its perimeter without any discernible break.

## References

[BIO024554C1] Bansil R. and Turner B. S. (2006). Mucin structure, aggregation, physiological functions and biomedical applications. *Curr. Opin. Colloid Interface Sci.* 11, 164-170. 10.1016/j.cocis.2005.11.001

[BIO024554C2] Bocchinfuso D. G., Taylor P., Ross E., Ignatchenko A., Ignatchenko V., Kislinger T., Pearson B. J. and Moran M. F. (2012). Proteomic profiling of the planarian Schmidtea mediterranea and its mucous reveals similarities with human secretions and those predicted for parasitic flatworms. *Mol. Cell. Proteomoics* 11, 681-691. 10.1074/mcp.M112.019026 PMC3434776 22653920

[BIO024554C3] Bowen I. D., Ryder T. A. and Winters C. (1975). The Distribution of Oxidizable Mucosubstances and Polysaccharides in the Planarian *Polycelis tenuis* Iijima. *Cell Tissue Res.* 161, 263-275. 10.1007/BF00220373 51685

[BIO024554C4] Denny M. W. (1989). Invertebrate mucous secretions: functional alternatives to vertebrate paradigms. *Symp. Soc. Exp. Biol.* 43, 337-366. 2701483

[BIO024554C5] Flammang P. (1996). Adhesion in echinoderms. In *Echinoderm Studies*, Vol. 5 (ed. Jangoux M. and Lawrence J. M.), pp. 1-60. Rotterdam: A. A. Balkema.

[BIO024554C6] Fudge D. S., Schorno S. and Ferraro S. (2015). Physiology, biomechanics, and biomimetics of hagfish slime. *Annu. Rev. Biochem.* 84, 947-967. 10.1146/annurev-biochem-060614-034048 25534639

[BIO024554C7] Greaves J. L. and Wilson C. G. (1993). Treatment of diseases of the eye with mucoadhesive delivery systems. *Adv. Drug Deliv. Rev.* 11, 349-383. 10.1016/0169-409X(93)90016-W

[BIO024554C8] Köwitsch A., Zhou G. and Groth T. (2017). Medical application of glycosaminoglycans - a review. *J. Tissue Eng. Regen. Med*. Epub ahead of print, doi:10.1002/term.2398 10.1002/term.2398 28079984

[BIO024554C9] Ludwig A. (2005). The use of mucoadhesive polymers in ocular drug delivery. *Adv. Drug Deliv. Rev.* 57, 1595-1639. 10.1016/j.addr.2005.07.005 16198021

[BIO024554C10] Martin G. G. (1978). A new function of rhabdites: mucus production for ciliary gliding. *Zoomorphologie* 91, 235-248. 10.1007/BF00999813

[BIO024554C11] Pedersen K. J. (1953). Some features of the fine structure and histochemistry of planarian subepidermal gland cells. *Cell Tissue Res.* 50, 121-142.

[BIO024554C12] Pedersen K. J. (1963). Sliime-secreting cells of planarians. *Ann. N. Y. Acad. Sci.* 106, 424-443. 10.1111/j.1749-6632.1963.tb16655.x 13942330

[BIO024554C13] Scott J. E. (1980). Collagen–proteoglycan interactions. Localization of proteoglycans in tendon by electron microscopy. *Biochem. J.* 187, 887-891. 10.1042/bj1870887 7188429 PMC1162476

[BIO024554C14] Silberberg A. and Meyer F. A. (1982). Structure and function of mucus. In *Mucus in health and disease II, Adv. Exp. Med. Biol.*, Vol. 144 (ed. Chantler E. N., Elder J. B. and Elstein M.), pp. 53-74. New York: Plenum Press. 10.1007/978-1-4615-9254-9_6 7044068

[BIO024554C16] Smith A. M. (2002). The structure and function of adhesive gels from invertebrates. *Integr. Comp. Biol.* 42, 1164-1171. 10.1093/icb/42.6.1164 21680401

[BIO024554C17] Smith A. M. and Morin M. C. (2002). Biochemical differences between trail mucus and adhesive mucus from marsh periwinkle snails. *Biol. Bull.* 203, 338-346. 10.2307/1543576 12480724

[BIO024554C18] Smith J. III, Tyler S., Thomas M. B. and Rieger R. M. (1982). The morphology of turbellarian rhabdites: phylogenetic implications. *Julian Trans. Am. Microsc. Soc.* 101, 209-228. 10.2307/3225810

[BIO024554C19] Smith A. M., Quick T. J. and St. Peter R. L. (1999). Differences in the composition of adhesive and non-adhesive mucus from the limpet *Lottia limatula*. *Biol. Bull.* 196, 34-44. 10.2307/1543164 25575383

[BIO024554C20] Tyler S. (1976). Comparative ultrastructure of adhesive systems in the turbellaria. *Zoomorphology* 84, 1-76. 10.1007/BF02568557

[BIO024554C21] Tyler S. (1988). The role of function in determination of homology and convergence - examples from invertebrate adhesive organs. *Fortschr. Zool.* 36, 331-347.

[BIO024554C22] Werneke S. W., Swann C., Farquharson L. A., Hamilton K. S. and Smith A. M. (2007). The role of metals in molluscan adhesive gels. *J. Exp. Biol.* 210, 2137-2145. 10.1242/jeb.006098 17562887

[BIO024554C23] Whittington I. D. and Cribb B. W. (2001). Adhesive secretions in the Platyhelminthes. *Adv. Parasitol.* 48, 101-224. 10.1016/S0065-308X(01)48006-7 11013756

[BIO024554C24] Wilks A. M., Rabice S. R., Garbacz H. S., Harro C. C. and Smith A. M. (2015). Double-network gels and the toughness of terrestrial slug glue. *J. Exp. Biol.* 218, 3128-3137. 10.1242/jeb.128991 26276864

[BIO024554C25] Yamada S., Sugahara K. and Özbek S. (2011). Evolution of glycosaminoglycans. *Comm. Int. Biol.* 4, 150-158. 10.4161/cib.4.2.14547 PMC3104567 21655428

